# Supplementary material for: An open-source framework for large-scale, flexible evaluation of biomedical text mining systems
Source: J Biomed Discov Collab. 2008 Jan 29;3:1. doi: 10.1186/1747-5333-3-1 (PMC2276192; doi:10.1186/1747-5333-3-1)
Supplement: Additional file 1 — Evaluation platform source code. The additional file contains the software evaluation framework discussed in this paper. The most current release can be downloaded from SourceForge.net [1]. See the accompanying README file for instructions on installation and use. Also included in the distribution is a collection of UIMA wrappers for some commonly used BioNLP tools and annotated corpora. [file 1747-5333-3-1-S1.zip › bionlp_uima_repository-1.0.1/docs/api/edu/uchsc/ccp/util/nlp/GenericTextUtils.html]

GenericTextUtils (Center for Computational Pharmacology Util/UIMA API)


|  |  |  |
| --- | --- | --- |
| **Overview** \| **Package** \| **Class** \| **Tree** \| **Deprecated** \| **Index** \| **Help** | |  |
| PREV CLASS   **NEXT CLASS** | **FRAMES**    **NO FRAMES**     **All Classes** |  |
| SUMMARY: NESTED \| FIELD \| CONSTR \| METHOD | DETAIL: FIELD \| CONSTR \| METHOD |  |


---


## edu.uchsc.ccp.util.nlp Class GenericTextUtils

```
java.lang.Object
  edu.uchsc.ccp.util.nlp.GenericTextUtils
```

---

``` public class GenericTextUtils extends Object ```

This class encompasses a collection of utilities for processing and dealing with text.

**Author:**
:   William A. Baumgartner, Jr.

---

| **Field Summary** | |
| --- | --- |
| `static String[]` | `STOPWORDS` |


**Constructor Summary**

`GenericTextUtils()`


| **Method Summary** | |
| --- | --- |
| `static int` | `determineDocumentSection(int spanStart, int[] sectionIDs, int[] sectionLengths)`             Determines the document section identifier for a given span based on the document section IDs and span lengths provided. |
| `static String` | `escapeCharacterForRegEx(String regex)`             Prepares a string to be used in a Regular Expression search. |
| `static String` | `exampleDocText()`             Returns some sample text |
| `static int` | `findFirstNonHTMLCharacter(String htmlText)` |
| `static int` | `findLastNonHTMLCharacter(String htmlText)` |
| `static List<TextAnnotation>` | `findTargetInText(String targetText, String originalText, int startLookingForMatchesAtIndex, String documentID, String classMentionType, Annotator annotator, boolean unconstrained)`             This method simple returns text annotations created from finding the target string within the original text string using regular expressions |
| `static HashMap<String,String>` | `getPennTreebankTagLabelLookup()`             Returns a HashMap containing the linguistic annotation tags used by the Penn Treebank, and their corresponding clause, phrase, or token labels.  Key = Tag, e.g. |
| `static Set<String>` | `getSetFromString(String setString)`             This method performs the reverse of the Set.toString() method. |
| `static String` | `getTokensAfter(int numTokens, int index, String documentText)` |
| `static String` | `getTokensBefore(int numTokens, int index, String documentText)` |
| `static boolean` | `isNumber(String possibleNumber)` |
| `static boolean` | `isStopWord(String word)` |
| `static XMLReader` | `makeXMLReader()`             Taken from: http://userpage.fu-berlin.de/~ram/pub/pub\_jf47hxhHHt/java\_sax\_parser\_en |
| `static PrintStream` | `openPrintStream(String outputFileLocation)`             Opens a PrintStream for printing to the given output file location |
| `static PrintStream` | `openPrintStream(String outputFileLocation, boolean append)`             Opens a PrintStream for printing to the given output file location |
| `static BufferedReader` | `openReader(String inputFileLocation)`             Opens a BufferedReader for reading from a text file |
| `static String` | `removeStopWords(String queryStr)`             Removes stop words from the input text string. |
| `static String` | `replaceXMLspecialCharacters(String line)`             Replaces XML special characters with their ASCII equivalents & --> &, etc. |
| `static ArrayList<KeyValuePair>` | `sortHashMapByValues(Map<String,Integer> inputMap)`             Sorts a Hashmap by the values contained in the HashMap |
| `static List<TextAnnotation>` | `tokenizeIntoSentences(String baseDirectory, String documentText, String documentID)`             This method tokenizes the input text into sentences via the KeX sentence tokenizing utility.    It should be noted that sometimes the KeX sentence tokenizer modifies the output text (usually by adding or deleting a space) so that it is not possible to determine the offset in the original text. |

**Methods inherited from class java.lang.Object**

`clone, equals, finalize, getClass, hashCode, notify, notifyAll, toString, wait, wait, wait`

**Field Detail**

### STOPWORDS

```
public static String[] STOPWORDS
```


**Constructor Detail**

### GenericTextUtils

```
public GenericTextUtils()
```


**Method Detail**

### openPrintStream

```
public static PrintStream openPrintStream(String outputFileLocation)
                                   throws IOException
```

:   Opens a PrintStream for printing to the given output file location

    :   **Parameters:**: `outputFileLocation` - path to the output file **Returns:**: an open PrintStream **Throws:**: `IOException`

---


### openPrintStream

```
public static PrintStream openPrintStream(String outputFileLocation,
                                          boolean append)
                                   throws IOException
```

:   Opens a PrintStream for printing to the given output file location

    :   **Parameters:**: `outputFileLocation` - path to the output file: `append` - **Returns:**: an open PrintStream **Throws:**: `IOException`

---


### openReader

```
public static BufferedReader openReader(String inputFileLocation)
                                 throws IOException
```

:   Opens a BufferedReader for reading from a text file

    :   **Parameters:**: `inputFileLocation` - path to the input file **Returns:**: an open BufferedReader **Throws:**: `IOException`

---


### exampleDocText

```
public static String exampleDocText()
```

:   Returns some sample text

    :   **Returns:**: a string of sample text

---


### sortHashMapByValues

```
public static ArrayList<KeyValuePair> sortHashMapByValues(Map<String,Integer> inputMap)
```

:   Sorts a Hashmap by the values contained in the HashMap

    :   **Parameters:**: `inputMap` - the HashMap to be sorted **Returns:**: an ArrayList containing sorted KeyValuePairs

---


### getSetFromString

```
public static Set<String> getSetFromString(String setString)
```

:   This method performs the reverse of the Set.toString() method. It reads in a string with format "[A,B,C]" and returns a Set with A, B,
    and C as members of the set.  
      
    NOTE: If the original set contained Strings with commas, then this method will be unreliable.

    :   **Parameters:**: `setString` - **Returns:**

---


### removeStopWords

```
public static String removeStopWords(String queryStr)
```

:   Removes stop words from the input text string. Stop words list taken from PUBMED.

    :   **Parameters:**: `queryStr` - the string to be processed **Returns:**: the input string without stop words

---


### isStopWord

```
public static boolean isStopWord(String word)
```

---


### tokenizeIntoSentences

```
public static List<TextAnnotation> tokenizeIntoSentences(String baseDirectory,
                                                         String documentText,
                                                         String documentID)
```

:   This method tokenizes the input text into sentences via the KeX sentence tokenizing utility.  
      
    It should be noted that sometimes the KeX sentence tokenizer modifies the output text (usually by adding or deleting a space) so that it is not
    possible to determine the offset in the original text. When this occurs an error is displayed to the screen.

    :   **Parameters:**: `baseDirectory` - - location of oneline program (part of KeX Entity Identifier): `documentText` - - text to be tokenized into sentences (and words are delimited by whitespace) **Returns:**: an ArrayList containing two ArrayLists, the first containing Strings where each String is a sentence, and the second containing Strings where each String is a word (token).

---


### determineDocumentSection

```
public static int determineDocumentSection(int spanStart,
                                           int[] sectionIDs,
                                           int[] sectionLengths)
```

:   Determines the document section identifier for a given span based on the document section IDs and span lengths provided.

    :   **Parameters:**: `spanStart` - the start of the span for the text whose document section ID is to be determined: `sectionIDs` - an int[] containing the document section IDs associated with a given document: `sectionLengths` - an int[] containing the lengths of each document section associated with a given document **Returns:**: The document section ID.

---


### escapeCharacterForRegEx

```
public static String escapeCharacterForRegEx(String regex)
```

:   Prepares a string to be used in a Regular Expression search. This method places a backslash in front of characters that need to be escaped in
    order to be recognized in a regular expression

    :   **Parameters:**: `regex` - the String to be converted into a Regular Expression search string **Returns:**: the converted String

---


### makeXMLReader

```
public static final XMLReader makeXMLReader()
                                     throws Exception
```

:   Taken from: http://userpage.fu-berlin.de/~ram/pub/pub\_jf47hxhHHt/java\_sax\_parser\_en

    :   **Returns:**: an XMLReader **Throws:**: `Exception`

---


### replaceXMLspecialCharacters

```
public static String replaceXMLspecialCharacters(String line)
```

:   Replaces XML special characters with their ASCII equivalents & --> &, etc.

    :   **Parameters:**: `line` - text to be converted **Returns:**: the converted text

---


### getPennTreebankTagLabelLookup

```
public static HashMap<String,String> getPennTreebankTagLabelLookup()
```

:   Returns a HashMap containing the linguistic annotation tags used by the Penn Treebank, and their corresponding clause, phrase, or token labels.  
    Key = Tag, e.g. "NP"; Value = "clause" "phrase" or "token"

    :   **Returns:**: a HashMap containing tag/label pairs where the tags are Penn Treebank linguistic annotation tags and the labels are "clause","phrase",or "token"

---


### findTargetInText

```
public static List<TextAnnotation> findTargetInText(String targetText,
                                                    String originalText,
                                                    int startLookingForMatchesAtIndex,
                                                    String documentID,
                                                    String classMentionType,
                                                    Annotator annotator,
                                                    boolean unconstrained)
```

:   This method simple returns text annotations created from finding the target string within the original text string using regular expressions

    :   **Parameters:**: `target` -: `originalText` -: `startLookingForMatchesAtIndex` - the index where we will start looking for matches within the original text: `documentID` - this is optional. If one if provided it will be used, if null is provided, -1 will be used.: `classMentionType` - this is also optional. If one is provided, a ClassMention of that type will be added to the TextAnnotation: `annotator` - this is also options. If one is provided, it is added to the TextAnnotation. If it is null, a default annotator is added instead. **Returns:**

---


### isNumber

```
public static boolean isNumber(String possibleNumber)
```

---


### findFirstNonHTMLCharacter

```
public static int findFirstNonHTMLCharacter(String htmlText)
```

---


### findLastNonHTMLCharacter

```
public static int findLastNonHTMLCharacter(String htmlText)
```

---


### getTokensBefore

```
public static String getTokensBefore(int numTokens,
                                     int index,
                                     String documentText)
```

---


### getTokensAfter

```
public static String getTokensAfter(int numTokens,
                                    int index,
                                    String documentText)
```


---


|  |  |  |
| --- | --- | --- |
| **Overview** \| **Package** \| **Class** \| **Tree** \| **Deprecated** \| **Index** \| **Help** | |  |
| PREV CLASS   **NEXT CLASS** | **FRAMES**    **NO FRAMES**     **All Classes** |  |
| SUMMARY: NESTED \| FIELD \| CONSTR \| METHOD | DETAIL: FIELD \| CONSTR \| METHOD |  |


---
